# Profiling and Structural Characterization of High Neu5Gc or Sulfate-Containing *O*-glycans from Hyla Rabbit Intestinal Mucin

**DOI:** 10.3390/molecules24071365

**Published:** 2019-04-07

**Authors:** Qianyun Fu, Guoyun Li, Chen Wang, Ya Wang, Qinying Li, Jiejie Hao, Guangli Yu

**Affiliations:** 1 Key Laboratory of Marine Drugs, Ministry of Education, Shandong Provincial Key Laboratory of Glycoscience and Glycotechnology, School of Medicine and Pharmacy, Ocean University of China, Qingdao 266003, China; fqy15764210034@163.com (Q.F.); wangchen_echo@163.com (C.W.); yawangouc@163.com (Y.W.); liqinying2010@163.com (Q.L.); 2009haojie@ouc.edu.cn (J.H.); glyu@ouc.edu.cn (G.Y.); 2 Laboratory for Marine Drugs and Bioproducts, Qingdao National Laboratory for Marine Science and Technology, Qingdao 266003, China

**Keywords:** Hyla rabbit intestinal mucin, *O*-glycan, Neu5Gc, structural characterization

## Abstract

Intestinal mucins constitute the major component of the mucus covering the epithelium of the gastrointestinal tract, thereby forming a barrier against microbial colonization. Rabbits are bred in large numbers worldwide, with little known about intestinal *O*-glycosylation despite this insight being crucial to the understanding of host-pathogen interactions. In the present study, a major mucin-type glycopeptide (RIF6) of hyla rabbit intestine was isolated and the *O*-glycans were extensively characterized based on liquid chromatography-tandem mass spectrometry (LC-MS/MS) combined with bioinformatics approaches. Thirty-three *O*-glycans were identified, and most of them were sulfated or sialylated glycans. It was worth noting that Neu5Gc-containing structures within sialylated *O*-glycans accounted for 91%, which were extremely different from that of other species including humans, mice, chickens, etc. Sulfated glycans accounted for 58%, unique disufated and sulfated-sialylated glycans were also detected in rabbit intestinal mucin. These structural characterization reflected species diversity and may provide deeper insights into explaining the adaptability of hyla rabbit to the environment.

## 1. Introduction

The intestine, an important part of the digestive system, is comprised of a complex organization of epithelium, immune cells, and resident microbiota [1]. The intestinal epithelium is covered by a mucus layer, which together with other substances forms the front line of defense to prevent microbial colonization and avoid erosion of the underlying epithelia [2].

Intestinal mucins, the major components of the mucus layer in intestine, are extensively *O*-glycosylated proteins that comprise up to 80% of the mucin by weight. These mucins play an essential role in protecting the underlying epithelium from chemical and mechanical stresses. Additionally, the mucin provides binding sites for pathogenic microbes, as well as serves as an abundant potential carbon source for resident microflora [3]. Several studies have indicated that alterations in mucin structure can be utilized to distinguish different species or certain diseases, such as Crohn’s disease, cystic fibrosis, or cancer [4,5,6,7,8,9,10,11]. Furthermore, *O*-glycan structural diversity in different species can contribute to the selection of microbial communities along and across the intestinal tract [12,13].

Rabbits are herbivores that are bred in large volumes worldwide. Infectious diseases of the digestive system currently cause up to 60% mortality of rabbit, which greatly influence rabbit farming. These enteric diseases are caused by the presence of the pathogen [14]. Previous studies have indicated that endogenous glycans, such as *O*-glycans, exert a great effect on intestinal health by maintaining the balance of microbial community [15]. However, despite the intestine being important to the overall health, little is known regarding unique intestinal *O*-glycosylation and how it impacts host-pathogen interactions in rabbits. Therefore, rabbit intestinal mucin-type glycopeptides were obtained and the *O*-glycans were characterized using LC-MS^n^ and various bioinformatics approaches in glycomics.

## 2. Results

### 2.1. Purification and Chemical Analysis of Hyla Rabbit Intestinal Mucins

Papain was used to obtain the mucin-type peptides from the rabbit intestines. Following purification by using QFF chromatography, four glycopeptide fractions (RIF4, RIF6, RIF8, and RIF10) were acquired. According to the monosaccharide composition analysis (Supplementary Information, Table S1), RIF6 was predominantly composed of Gal, GlcNAc, GalNAc and Sialic acid (Supplementary Information, Figure S1), which was consistent with mucin character, and thus was further characterized for the *O*-glycan studies. Whereas minor content of GalNAc and Gal in RIF4 ruled out its possibility of being *O*-glycans, RIF8 and RIF10 were glycosaminoglycans according to their monosaccharide composition.

### 2.2. Structural Analysis of O-glycan by LC-MS/MS

The *O*-glycans were released by using the reductive β-elimination method, and all *O*-glycans would be existed in its alditol form. The profiling of the *O*-glycans was performed on a porous graphitized carbon column separation and online LC-MS^n^ analysis technique [16]. All data obtained from LC-MS^n^ were analyzed with bioinformatics softwares. Collision-induced dissociation (CID), a fragmentation technique, was adopted to characterize the fine structures of *O*-glycan. The parent ions derived from the full MS were labeled as various fragments which could then be utilized to determine the core type, specific linkage mode, and the positions of fucose or sialic acid [17,18,19,20,21]. The naming rules for the fragment ions were based on previously described nomenclature [22], with A_i_, B_i_, and C_i_ representing fragment ions containing non-reducing ends, while X_j_, Y_j_, and Z_j_ representing fragment ions containing reducing ends. Additionally, the subscript α is used to represent longer branches in the *O*-glycan chain, while the subscript β represents shorter ones.

#### 2.2.1. LC−MS/MS Revealed Overall Structural Characteristics

**Figure 1:**
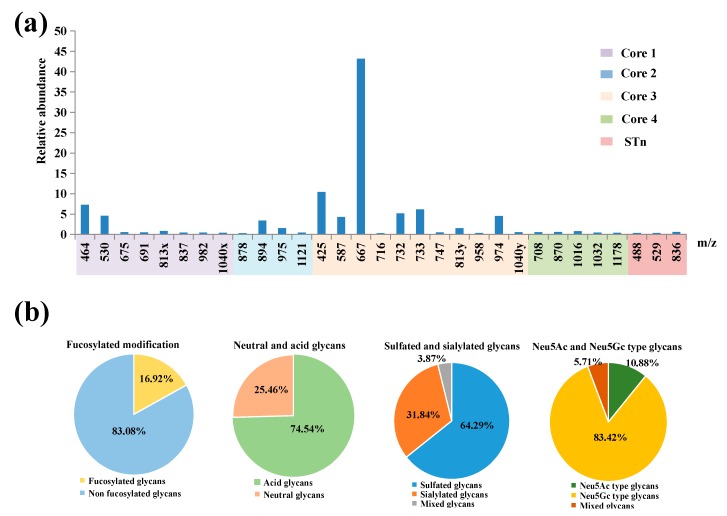
Composition and content of *O*-glycans. (**a**) Quantitative graph of 33 kinds of *O*-glycans existed in rabbit intestines. (**b**) Percentage of different types of *O*-glycans with various modifications.

*O*-Glycans from RIF6 were detected as singly and doubly charged ions in negative-ion mode (Table 1). Overall, 33 *O*-glycans were characterized, containing six sulfated or Neu5Gc-containing structures unique to the rabbits (*m*/*z* 747, 837, 982, 488, 529, 836). The *O*-glycans were 2–6 residues long, and most were sulfated or sialylated. The distribution of *O*-glycans from RIF6 was 25.46% neutral, 10.40% sialylated, 57.7% sulfated, 16.92% fucosylated and 4.87% sulfated-sialylated glycans (Figure 1). It is worth noting that the five most abundant structures (*m*/*z* 667, 425, 464, 733 and 732) accounted to 72.22% of total content. Most of these five structures were sialylated or sulfated core 3.

#### 2.2.2. Core 3 Extended with Type II LacNAc Is the Main *O*-glycan Type

**Figure 2:**
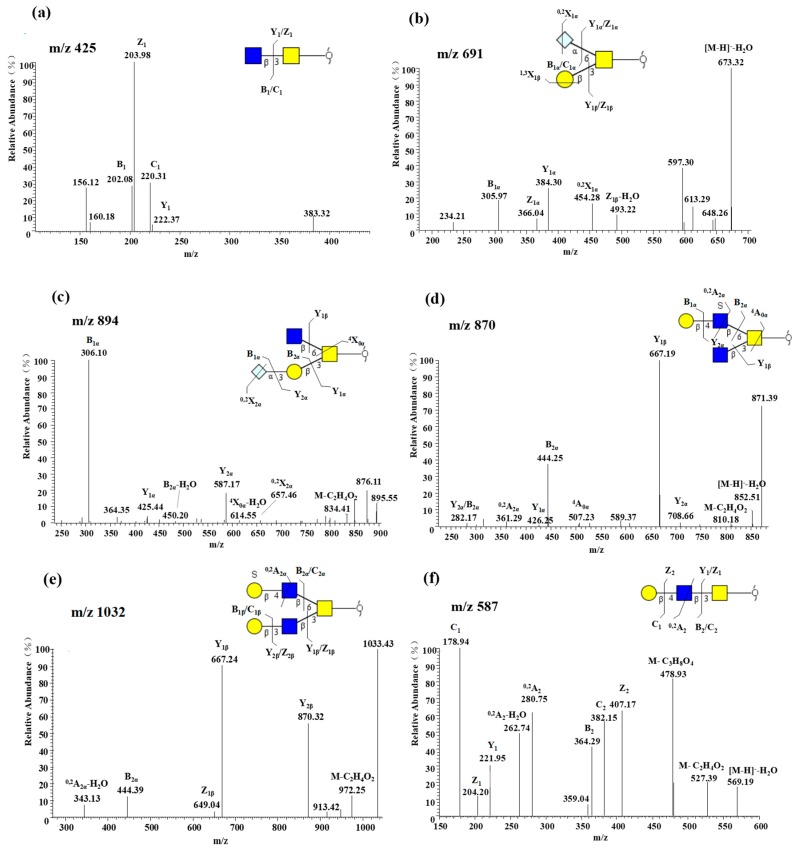
MS/MS spectra in the negative ion mode and proposed fragmentation of *O*-glycan with different core types and two isomeric oligosaccharides: (**a**) core 3 oligosaccharide at *m*/*z* 425 [GlcNAcβ1-3GalNAc-ol]; (**b**) core 1 oligosaccharide at *m*/*z* 691 [Galβ1-3(Neu5Gcα2-6)GalNAc-ol]; (**c**) core 2 oligosaccharide at *m*/*z* 894 [Neu5Gcα2-3Galβ1-3(GlcNAcβ1-6)GalNAc-ol]; (**d**) core 4 oligosaccharide at *m*/*z* 870 [Galβ1-4(SO_3_^−^)GlcNAcβ1-6(GlcNAcβ1-3)GalNAc-ol]; (**e**) oligosaccharide with both Galβ1-3 linked and Galβ1-4 linked to a GlcNAc residue; (**f**) oligosaccharide with a Galβ1-4 linked to a GlcNAc residue.

Core structures can be easily identified from CID spectra by the loss of a moiety from the pseudomolecular ion [16,20,23]. Core 3 structure represented the largest proportion of glycans (75.22%) based on the integral areas of the MS analysis. The B type ion (*m*/*z* 202) in Figure 2a and Z_2_ (*m*/*z* 407) in Figure 2f indicate the presence of core 3 type structures. Furthermore, core 1 can be also detected based on different Z_1β_ ions formed by the loss of the Gal (Figure 2b). Two structures (*m*/*z* 813, 1040) containing both core 1 and core 3 isomers are also detected. The *m*/*z* 614 and *m*/*z* 507 fragments (Figure 2c,d) indicate the presence of cores 2 and 4. In Figure 2f,e, the ^0,2^A_2_/^0,2^A_2α_-H_2_O fragment ion at *m*/*z* 280, *m*/*z* 343 indicates the presence of a type II chain. The majority of identified glycans were type II lactosamine structures (15 of 33), accounting for 64.59% according to integral area. Among these structures, core 3 structures were the main portion elongated with a type II lactosamine.

#### 2.2.3. Notable Feature of High Neu5Gc-containing *O*-glycans

**Figure 3:**
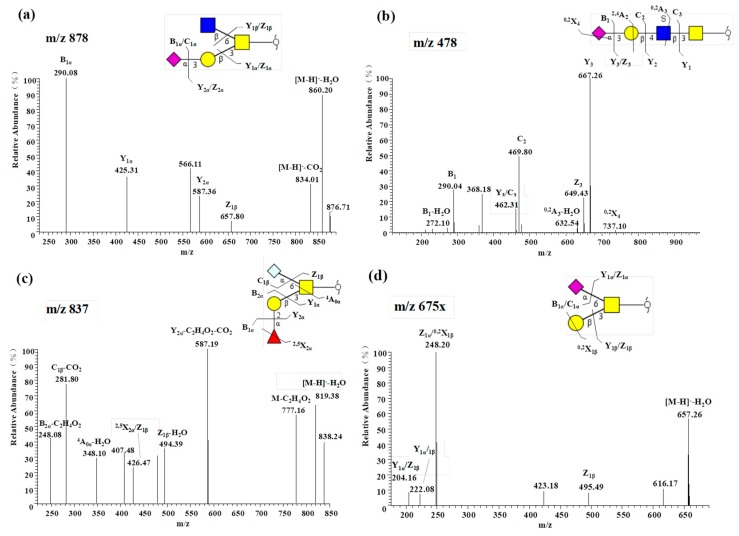
MS/MS spectra of sialylated oligosaccharides recorded in the negative ion mode: (**a**) and (**b**) oligosaccharide with a Neu5Acα2-3 linked to a Gal residue; (**c**) oligosaccharide with a Neu5Gcα2-6 linked to a GalNAc-ol; (**d**) oligosaccharide with a Neu5Acα2-6 linked to a GalNAc-ol.

*O*-glycans from RIF6 were heavily sialylated. Almost half of the identified *O*-glycans (16 of 33) were sialylated with 18.18% sialylated core 1, 6.06% sialylated core 2, 15.15% sialylated core 3 and 9.09% sialylated Tn antigen. Two types of sialic acid, Neu5Ac (6 of 33) and Neu5Gc (11 of 33), were detected. Herein, the B ion at *m*/*z* 290 in MS/MS spectra (Figure 3a,b) corresponds to a Neu5Ac residue and the ion at *m*/*z* 306 (Figure 2b,c) corresponds to a Neu5Gc residue. One structure at *m*/*z* 982 in the MS/MS spectra (Supplementary Information, Figure S3) was also found containing Neu5Gc and Neu5Ac. Among these sialylated *O*-glycans, the Neu5Gc-containing glycans represented the most notable content and constituted 91%. The linkages included α2-3 linkages to Gal, α2-6 linkages to GalNAc-ol, or an α2-8 linkage to di- or polysialyl motifs. The fragment ion at *m*/*z* 469 (Figure 3b) serves as a diagnostic ion for a Neu5Ac residue directly linked to Gal via an α2-3 linkage. In addition to these ions, the fragment ion at *m*/*z* 348 (Figure 3c) is annotated as ross-ring cleavage of ^4^A_0_-H_2_O, indicating a sialic acid linkage to the core GalNAc-ol. The structure at *m*/*z* 675 contained two isomers with Neu5Ac linking to Gal (Supplementary Information, Figure S3) or core GalNAc-ol (Figure 3d). Seven of 16 sialylated structures contained α2-6 and six of 16 contained α2-3 linkages. Notably, 2 Neu5Gc-containing structures (*m*/*z* 894, 974) accounted for 56% according to the integral area.

#### 2.2.4. Characterization of Highly Sulfated *O*-glycans

**Figure 4:**
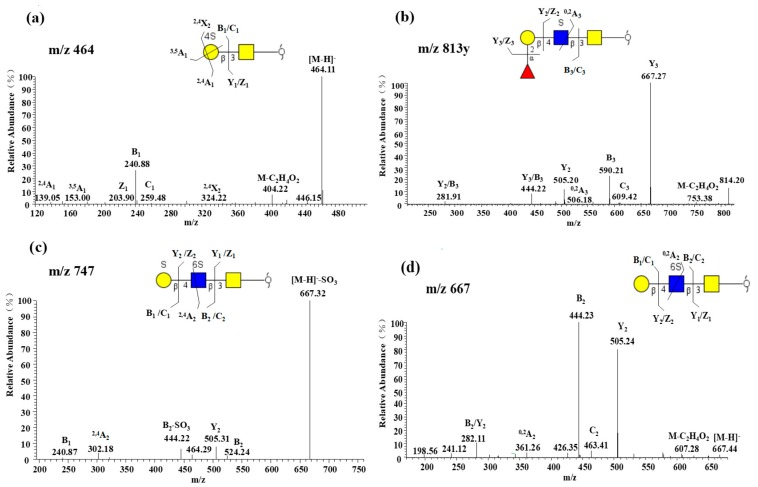
MS/MS spectra of different sulfated oligosaccharides recorded in the negative ion mode: (**a**) (SO_3_^−^)4Galβ1-3GalNAc-ol; (**b**) Fucα1-2Galβ1-4(SO_3_^−^)GlcNAcβ1-3GalNAc-ol; (**c**) (SO_3_^−^)Galβ1-4 (SO_3_^−^)6GlcNAcβ1-3GalNAc-ol; (**d**) Galβ1-4(SO_3_^−^)6GlcNAcβ1-3GalNAc-ol.

*O*-glycans of RIF6 were highly sulfated with 15 of 33 *O*-glycans being identified. Among these sulfated glycans, most were monosulfated (14 of 15), with minor trace of disulfated glycans (Figure 4c). The content of sulfated glycans was up to 58% with one particular structure (*m*/*z* 667) accounting for 43%. The location of sulfate was found on Gal, GlcNAc or GalNAc. The presence of the *m*/*z* 241 (Figure 4a) or *m*/*z* 282 (Figure 4b) ions indicates that the sulfate groups are located on a Hex or HexNAc. The C-4 position linked to a Gal and the C-6 to GlcNAc were detected. A sulfated linkage to a monosaccharide residue can be distinguished by various specific ring cleavages [17]. Herein, the ^2,4^A and ^3,5^A fragment ions (Figure 4a) indicate a substitution of C-4 linkage. The fragment ion generated via ^0,4^A-type and ^0,2^A-type cleavage (Figure 4d) can serve as the diagnostic ion for the C-6 sulfate substitution of HexNAc.

#### 2.2.5. Presence of α1-2 Fucose-containing *O*-glycans

**Figure 5:**
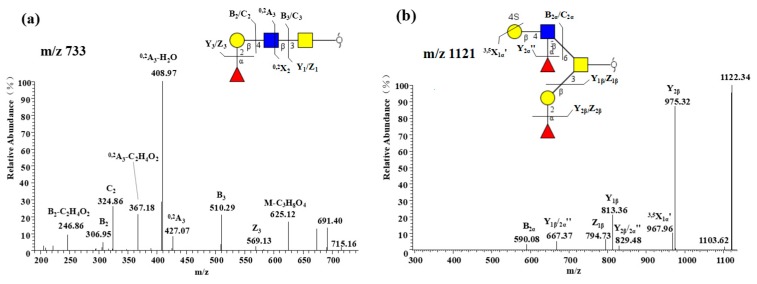
MS/MS spectra of two fucosylated oligosaccharides recorded in the negative ion mode: (**a**) oligosaccharide with α1-2 fucose linkage to Gal residue; (**b**) oligosaccharide with α1-2 fucose linkage to Gal residue and α1-3 fucose linkage to GlcNAc residue.

In mucin-type *O*-glycans, the location of fucose can, to a certain extent, determine the type of epitope [20]. In this study, 12 of 33 structures contained fucose residues, mainly with an α1-2 fucose linkage to Gal to form several antigens. Among these antigens, the blood group H epitopes (10 of 33) were predominated, along with Lewis^x^ (1 of 33) and group A (2 of 33). The total level of fucosylated glycans constituted 17% of all glycans base on MS intensities (Figure 1). The content of H epitopes was up to over 86% within glycans with fucose residues. Non-reducing end H antigen fragments are always accompanied by the B-C_2_H_4_O_2_ ions of *m*/*z* 247 (Figure 5a), which is characteristic of a C-2 substituted Gal. In Figure 5b, the Y_2β_ (*m*/*z* 975) and Y_1β_ (*m*/*z* 813) cleavage ions indicate that an α1-2 fucose linkage to Gal was present.

## 3. Discussion

*O*-Glycans on intestinal mucin are of great significance in protecting against harmful substances, maintaining balance of beneficial microbial community and helping rabbits to keep healthy. In this study, *O*-glycans from rabbit intestinal mucin-type glycopeptides were characterized, rendering this the first elaborate *O*-glycan profiling of hyla rabbit mucins. 33 *O*-glycans were characterized, and most were heavily sialylated or sulfated. These identified *O*-glycans showed great similarity (17 of 33) to that of pigs, which are also domestic mammal as rabbits (Supplementary Information, Table S2). Additionally, one disulfated (*m*/*z* 747) and five Neu5Gc-containing structures (*m*/*z* 488, 529, 836, 837, 982) were unique to the rabbits. This reflects the joint action of genetic inheritance and environmental selectivity.

Cores 1, 2, 3, 4 and several Tn antigen were detected. Of these glycans, core 3 was the most abundant core type. This structure has showed an important role in reducing intestinal permeability and levels of bacteria within mucosa, thus promoting mucosal barrier function [24]. These findings are similar to previous studies in human [19] and chicken [25] intestines. However, it shows differences in other species, such as the porcine colon containing predominantly core 4 [26], mouse intestines mostly comprising core 2 [27], and fish intestine mostly consisting of core 5 [28]. Particularly, *m*/*z* 667, a sulfated core 3 structure, made up 43.19% of total glycans, which extensively existed in the intestines of humans and chickens with same core structure, but displayed in mouse and pig intestines with different isomers. Species-specific core type glycan expression reflects differences in needs of intestinal microorganism, encountered environmental challenges, and species diversity.

Abundant Neu5Gc-containing glycans was the most unique feature in rabbit intestines. The identified Neu5Gc-containing structures were never found in intestines of humans or chickens whereas displayed 3 same glycans with pigs (Supplementary Information, Table S2). Notably, the content of Neu5Gc-containing glycans within sialylated *O*-glycans accounted for 91%, which was extremely higher than that in other species’ intestines. The most abundant three Neu5Gc-containing glycans (*m*/*z* 732, 974, 894) were only detected a small amount (1.8%) in pig intestines, but reached up to 13.08% of total content in the rabbit intestines. Interestingly, the content of Neu5Gcα2-3Gal structures in rabbit intestines accounted for 56% among sialylated glycans, even though only two structures (*m*/*z* 894, 974) were characterized. As prominent outermost carbohydrates on mucins, sialic acids play important regulatory and protective roles in cell biology [29]. Unique content of Neu5Gc may also alter the functions of endogenous receptors for sialic acids in the immune system [30]. Besides, many major pathogens gain access to their mammalian hosts by binding to certain sialic acids types or linkages in the surface of glycan chains. Previous studies have showed *E. coli* K99, a pathogen that has a strong preference for Neu5Gc, can cause serious diarrheal diseases in farm animals like cows and pigs [30]. Human influenza A virus preferentially recognize SAα2-6Gal linkage, whereas most animals are infected by α2,3-specific influenza A virus [31]. Another research revealed recognition of Neu5Gcα2-3Gal structure is related to the efficient replication of influenza viruses in duck intestines [32]. Therefore, abundant Neu5Gc-containing glycans may make rabbits be susceptible to diseases such as influenza viruses or diarrheal. Additionally, rabbits share less similar microorganisms composition with other species which are high in Neu5Ac, thus conferring protection for both of species from animal pathogens and diseases. For example, rabbits may be resistant to parasite infection which is in specificity toward Neu5Ac in rat intestines [33]. This parallels the loss of Neu5Gc in humans and chickens, which are the only two animals that lack the cytidine monophosphate (CMP)-*N*-acetylneuraminic acid hydroxylase (CMAH) gene required to synthesize Neu5Gc [34,35]. The absence of Neu5Gc makes humans and chickens be not prone to infections by viruses and bacteria which recognize Neu5Gc only and can live close to rabbits [36]. Rabbit intestines have been used as food source in many Asian countries, however, some studies have revealed that much intake of Neu5Gc-containing food may contribute to a higher cancer frequency and other dietary associated diseases in humans [37]. Thus, the higher rabbit intestinal Neu5Gc levels may also provide dietary references to prevent cancer or other related diseases in humans.

The extent of sulfation is further emphasized as another unique feature when compared with other results from intestines in human, pig and fish (Supplementary Information, Table S2). Fifteen out of 33 indentified *O*-glycans were sulfated structures in rabbit intestines. It is to be noted that the content of these sulfated glycans accounted for 58%. And most were monosulfated, which was consistent with the previous human mucin studies [38]. Compared with other species, one disulfated glycan (*m*/*z* 747) and two sulfated-sialylated glycans (*m*/*z* 958, 974) were also detected as unique structural features in rabbit intestines (Supplementary Information, Table S2). Sulfated structures in rabbit intestines were extended cores 1, 2, 3, 4, which were different from structures in other species, such as the absence of sulfated core 4 glycans in humans, even the absence of sulfated structures verified in fish intestines (Supplementary Information, Table S2). As was observed in rabbits, many species have extensively sulfated intestinal mucin *O*-glycans [25,27,39]. Previous studies showed that core 1–4 and 6 serve as potential substrates for sulphotransferases (STs). Two main mucin STs, GlcNAc6ST and Gal3ST, transfer sulphate from 3-phosphoadenosine 5-phosphosulphate (PAPS) to the 6-position of GlcNAc and the 3-position of Gal, respectively [40]. The expression of GlcNAc6ST and Gal3ST differ in different species. For instance, the GlcNAc6ST-2 transferase has been shown to be a major sulfation enzyme in the murine colon, and this enzyme is responsible for the predominant GlcNAc-6-*O*-sulfation in mouse colonic mucins [41]. The GlcNAc6ST-3 was mainly expressed in human intestines [42]. Additionally, Gal3ST-2 transferase was upregulated in the pathogen resistant chickens [25]. These expression changes of STs may account for various sulfated modification in intestinal mucins of different species. In another study, infected pig colons had lower levels of sulfated structures when compared with non-infected porcine [26]. Abundant charged structures (sulfated, sialylated and sulfated-sialylated) in rabbit intestines confer acidic properties which can change epithelial cell interactions or even modulate bacterial interactions by hindering bacterial degradation as previous studies have revealed [43,44].

Terminal fucosylated epitopes have been reported to act as binding sites for various pathogens. *Campylobactor jejuni*, the most common source of food poisoning and a common cause of death [45,46], is a bacterium usually present in the intestinal tract. Previous study reported the ABO blood group antigens can be utilized by *C. jejuni* as adhesion receptors [47]. The blood group H epitopes (10 of 33) may act as bacterial receptors within the rabbit intestine. However, this effect could be modulated when these structures carry charged residues, such as sialic acid or sulfate [25]. Large portions of the antigenic epitopes were modified by Neu5Gc or the addition of sulfate (Table 1), which may balance the relationship between intestines and bacteria, thus promoting the health of rabbits.

The analysis of rabbit intestinal mucin-type glycans provides a platform for deeper understanding on how structural differences in glycosylation could explain the adaptability to the environment. The structural features of rabbit intestines, especially the presence of highly sialylated and sulfated modification, may provide unique mucus environments to help specialized microflora to live and protect mucus from degradation. Notable and abundant content of Neu5Gc-containing glycans in rabbit intestines explains susceptibility or resistance to certain pathogens or diseases for rabbits and also provides dietary references for humans.

## 4. Materials and Methods

### 4.1. Materials and Chemicals

Hyla rabbit intestines were provided by Kangda Food Co., Ltd. (Qingdao, China). Papain was purchased from Amresco (Solon, OH, USA). Q-Sepharose Fast Flow resin was procured from GE Healthcare (Uppsala, Sweden). Mannose (Man), *N*-acetylglucosamine (GlcNAc), glucuronic acid (GlcA), galacturonic acid (GalA), *N*-acetylgalactosamine (GalNAc), glucose (Glc), galactose (Gal), xylose (Xyl), and fucose (Fuc), cetylpyridinium chloride (CPC), 1-phenyl-3-methyl-5-pyrazolone (PMP) and sodium borohydride (NaBH_4_) were purchased from Sigma-Aldrich (St. Louis, MO, USA). The Eclipse XDB-C18 column (4.6 mm × 150 mm, 5 μm) was obtained from Agilent (Santa Clara, CA, USA), and Hypercarb KAPPA Capillary Column (100 mm × 0.5 mm, 3 μm) was obtained from Thermo Fisher Scientific (Waltham, MA, USA). Sodium hydroxide (NaOH), trifluoroacetic acid (TFA), *N*-acetylneuraminic acid (Neu5Ac), *N*-glycolylneuraminic acid (Neu5Gc), and 1,2-diamino-4,5-methylenedioxybenzene (DMB derivative) were purchased from Aladdin (Shanghai, China).

### 4.2. Purification and Chemical Compositions of Rabbit Intestinal Mucin-Type Glycopeptides

The hyla rabbit intestines mucosa were degreased by adding 20 volumes of chloroform/methanol (2:1) for 15 h. The dried degreased mucosa powders were treated with papain, and the crude mucin-type glycopeptides were separated by cetylpyridinium chloride (CPC) precipitation. The nucleic acids that existed in the crude mucin-type glycopeptides were further removed by the isoelectric point precipitation method. The pure mucin-type glycopeptide (RIF6) was finally purified on a Q-Sepharose Fast Flow (QFF) column as previously described [48]. The purified fractions were dialyzed and lyophilized.

The monosaccharide composition was determined by using PMP labeling in conjunction with high-performance liquid chromatography (HPLC) as described by Chen et al. [49]. The sialic acid content was performed using a pre-column DMB derivatization as previous studies have reported [50].

### 4.3. O-Linked Glycans Released from RIF6

*O*-linked glycans were released from the hyla rabbit intestinal mucin-type glycopeptide by β-elimination. Briefly, the samples (0.5 mg) were incubated with 50 mM NaOH and 0.5 M NaBH_4_ at 50 °C for 14 h. Reactions were quenched with glacial acetic acid, 3 volumes of ethanol was added and then centrifuged, and the supernatants were dialyzed and freeze-dried.

### 4.4. Analysis of O-glycans Released from RIF6 by Liquid Chromatography-Mass Spectrometry (LC-MS/MS)

Herein, the LTQ-Orbitrap XL and Agilent 1260 capillary liquid systems were utilized for LC-MS/MS analysis. The *O*-glycan samples were separated using a Hypercarb KAPPA column maintained at 25 °C. The mobile phase A consisted of acetonitrile and the mobile phase B consisted of 10 mM ammonium bicarbonate. A loading volume of 0.2 µL was utilized at a flow rate of 8 µL·min^−1^ under the following gradient: 2%–8% A in 20 min; 8%–15% A in 50 min; 15%–35% A in 80 min; 35%–60% A in 50 min; and a final hold step from 60%–75% A in 5 min. Electrospray ionization-mass spectrometry (ESI-MS) was performed in negative ion polarity mode with an electrospray voltage of 3 kV, a capillary voltage of −41 V, a lens voltage of −120 V, and a capillary temperature of 275 °C. The instrument was operated in Fourier transform (FT) mode with a sheath gas flow rate of 8 L·min^−1^ and a mass range of 280–3000 *m*/*z*. A full MS scan was performed first, followed by a data-dependent collision-induced dissociation (CID) MS/MS scan of the 5 most abundant ions. To obtain optimal fragmentation, the normalized collision energy was 30 V.

### 4.5. O-glycan Structural Annotation and Assignments

The LC-MS/MS data was processed manually using Xcalibur (Thermo Fisher Scientific, Waltham, MA, USA) and GlycoWorkbench softwares (Alessio Ceroni et al., Imperial College, London, UK). Glycans were annotated manually based on their MS/MS spectra and then validated using available structures in the CarbBank database. The MS spectra and MS/MS spectra were analyzed using the Xcalibur software and the precise glycan structures were generated using GlycoWorkbench based on the different fracture fragments of the parent ions [51]. All of the quantitative data were calculated based on the integrated peak areas obtained from LC−MS chromatograms and formatted as a percentage (%) to represent relative quantitation.

## Figures and Tables

**Table 1:** The assigned *O*-glycans of RIF6 based on ESI-CID-MS/MS.

**Core 1**					
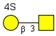	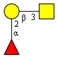	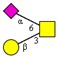		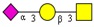	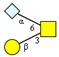
*m*/*z* 464	*m*/*z* 530	*m*/*z* 675x		*m*/*z* 675y	*m*/*z* 691
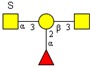	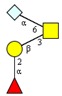			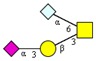	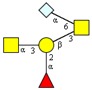
*m*/*z* 813x	*m*/*z* 837			*m*/*z* 982	*m*/*z* 1040 x
**Core 2**					
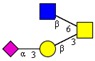	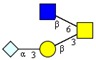	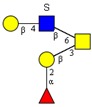		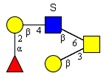	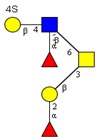
*m*/*z* 878	*m*/*z* 894	*m*/*z* 975x		*m*/*z* 975y	*m*/*z* 1121
**Core 3**					
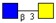	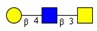	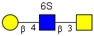	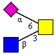	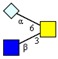	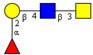
*m*/*z* 425	*m*/*z* 587	*m*/*z* 667 (333)	*m*/*z* 716	*m*/*z* 732	*m*/*z* 733
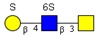	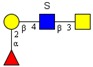	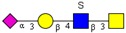		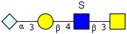	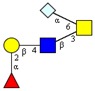
*m*/*z* 747 (373)	*m*/*z* 813y	*m*/*z* 958 (478)		*m*/*z* 974	*m*/*z* 1040 y (519)
**Core 4**					
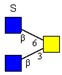	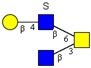	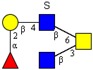		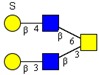	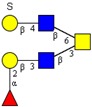
*m*/*z* 708	*m*/*z* 870	*m*/*z* 1016		*m*/*z* 1032	*m*/*z* 1178
**Other special structures**					
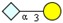		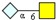		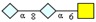	
*m*/*z* 488 (peeling)		*m*/*z* 529 (STn*)		*m*/*z* 836 (STn*)	

‘STn’: Sialyl-Tn antigen. Monosaccharide symbols are based on the Consortium for Functional Glycomics (CFG) nomenclature: blue square, GlcNAc; yellow square, GalNAc; yellow circle, Gal; purple diamond, Neu5Ac; write diamond, Neu5Gc; red triangle, Fuc.

## Supplementary Materials

The following are available online. Table S1: Monosaccharide composition of four fractions purified on a QFF column, Table S2: *O*-glycan structures assigned by ESI-CID-MS/MS in RIF6 and comparison with other species, Figure S1: HPLC Chromatogram for sialic acid standards solution (Neu5Ac, Neu5Gc) and sample (RIF6), Figure S2: ESI-CID-MS/MS spectra of the remaining *O*-glycans from RIF6, Figure S3: Total ion chromatogram of assigned *O*-glycan structures of RIF6.
