# Supplementary material for: Profiling and Structural Characterization of High Neu5Gc or Sulfate-Containing O-glycans from Hyla Rabbit Intestinal Mucin
Source: Molecules. 2019 Apr 7;24(7):1365. doi: 10.3390/molecules24071365 (PMC6480446; doi:10.3390/molecules24071365)
Supplement: Supplementary file 1 [file molecules-24-01365-s001.pdf]

Supplementary information

Profiling and Structural Characterization of High Neu5Gc or Sulfate-containing *O*-glycans from Hyla Rabbit Intestinal Mucin

Qianyun Fu [1], Guoyun Li [1,2*], Chen Wang [1], Ya Wang [1], Qinying Li [1], Jiejie Hao [1,2], Guangli Yu [1,2]

1 Key Laboratory of Marine Drugs, Ministry of Education, Shandong Provincial Key Laboratory of Glycoscience and Glycotechnology, School of Medicine and Pharmacy, Ocean University of China, Qingdao 266003, China; fqy15764210034@163.com (Q.F.); wangchen_echo@163.com (C.W.); yawangouc@163.com (Y.W.); liqinying2010@163.com (Q.L.); 2009haojie@ouc.edu.cn (J.H.); glyu@ouc.edu.cn (G.Y.).

2 Laboratory for Marine Drugs and Bioproducts, Qingdao National Laboratory for Marine Science and Technology, Qingdao 266003, China; liguoyun@ouc.edu.cn (G.L.); 2009haojie@ouc.edu.cn (J.H.); glyu@ouc.edu.cn (G.Y.).

* Correspondence: liguoyun@ouc.edu.cn; Tel.: +86-532-8203 1615

Received: date; Accepted: date; Published: date

Table S1. Monosaccharide composition of four fractions purified on a QFF column

Fraction	Monosaccharide composition (molar ratio)								
	Man	GlcNAc	GalNAc	GlcA	GalA	Glc	Gal	Rha	Fuc
RIF4	ND*	13.91	3.43	0.30	0.36	1.00	3.90	0.32	0.89
RIF6	ND*	69.88	52.40	2.70	ND*	1.00	42.86	ND*	6.58
RIF8	ND*	2.50	5.42	0.82	ND*	1.00	ND*	ND*	ND*
RIF10	22.49	2.78	174.08	64.08	ND*	1.00	5.49	0.32	ND*

* "ND"：Not detected.

Table S2. ***O*****-glycan structures assigned by ESI-CID-MS/MS in RIF6 and comparison with other species.**

m/z	Proposed Structures	Core type	Relative Abundance(%)	Humans	Chickens	Pigs	Mice	Fishes
			Neutral O-glycans					
425.1712^-	GlcNAcβ1-3GalNAcol	Core 3	10.42	√	√	×	√	√
530.2014^-	Fucα1-2Galβ1-3GalNAcol	Core 1	4.60	×	×	√	√	×
587.2213^-	Galβ1-4GlcNAcβ1-3GalNAcol	Core 3	4.30	*	√	√	*	√
733.2773^-	Fucα1-2Galβ1-4GlcNAcβ1-3GalNAcol	Core 3	6.15	*	*	√	*	*
			Sulfate-containging O-glycans					
464.1010^-	(SO_3^-)4Galβ1-3GalNAcol	Core 1	7.30	×	√	×	×	×
667.1766^-	Galβ1-4(SO_3^-)6GlcNAcβ1-3GalNAcol	Core 3	43.19	√	*	*	*	×
708.2025^-	(SO_3^-)GlcNAcβ1-6(GlcNAcβ1-3)GalNAcol	Core 4	0.55	×	×	√	×	×
747.1322^-	(SO_3^-)Galβ1-4(SO_3^-)6GlcNAcβ1-3GalNAcol	Core 3	0.52	×	×	×	×	×
813.2323^-	(SO_3^-)GalNAcα1-3(Fucα1-2)Galβ1-3GalNAcol	Core 1	2.39	*	*	√	*	×
	Fucα1-2Galβ1-4(SO_3^-)GlcNAcβ1-3GalNAcol	Core 3		√	*	√	*	×
870.2523^-	Galβ1-4(SO3-)GlcNAcβ1-6(GlcNAcβ1-3)GalNAcol	Core 4	0.61	×	*	*	*	×
975.2650^-	Fucα1-2Galβ1-4(SO_3^-)GlcNAcβ1-6(Galβ1-3)GalNAcol	Core 2		*	*	√	√	×
	Galβ1-4(SO_3^-)GlcNAcβ1-6(Fucα1-2Galβ1-3)GalNAcol	Core 2	1.54	*	*	√	*	×

1016.3102⁻	GlcNAcβ1-3[Fucα1-2Galβ1-4(SO_3^-)GlcNAcβ1-6]GalNAcol	Core 4	0.80	×	×	√	×	×
1032.3019⁻	(SO_3^-)Galβ1-4GlcNAcβ1-6(Galβ1-3GlcNAcβ1-3)GalNAcol	Core 4	0.20	×	×	×	*	×
1121.3380⁻	(SO_3^-)4Galβ1-4(Fucα1-3)GlcNAcβ1-6(Fucα1-2Galβ1-3)GalNAcol	Core 2	0.18	×	×	*	*	×
1178.3591⁻	(SO_3^-)Galβ1-4GlcNAcβ1-6(Fucα1-2Galβ1-3GlcNAcβ1-3)GalNAcol	Core 4	0.41	×	×	*	*	×
	Neu5Gc-containing O-glycans							
488.1658⁻	Neu5Gcα2-3Galol	peeling	0.37	×	×	×	×	×
529.1804⁻	Neu5Gcα2-6GalNAcol	STn	0.34	×	×	×	×	×
691.2303⁻	Galβ1-3(Neu5Gcα2-6)GalNAcol	Core 1	0.22	×	×	√	×	√
732.2565⁻	GlcNAcβ1-3(Neu5Gcα2-6)GalNAcol	Core 3	5.16	×	×	√	×	*
836.3022⁻	Neu5Gcα2-8Neu5Gcα2-6GalNAcol	STn	0.59	×	×	×	×	×
837.3053⁻	Fucα1-2Galβ1-3(Neu5Gcα2-6)GalNAcol	Core 1	0.18	×	×	×	×	×
894.3061⁻	Neu5Gcα2-3Galβ1-3(GlcNAcβ1-6)GalNAcol	Core 2	3.39	×	×	√	×	*
1040.3623⁻	GalNAcβ1-3(Fucα1-2)Galβ1-3(Neu5Gcα2-6)GalNAcol	Core 1	0.39	×	×	×	*	*
	Fucα1-2Galβ1-4GlcNAcβ1-3(Neu5Gcα2-6)GalNAcol	Core 3	0.28	×	×	×	*	*
	Neu5Ac-containing O-Glycans							
675.2558⁻	Galβ1-3(Neu5Acα2-6)GalNAcol	Core 1	0.27	√	×	√	×	√
	Neu5Acα2-3Galβ1-3GalNAcol	Core 1		√	×	√	√	√
716.2616⁻	GlcNAcβ1-3(Neu5Acα2-6)GalNAcol	Core 3	0.32	×	×	√	×	√
878.3114⁻	Neu5Acα2-3Galβ1-3(GlcNAcβ1-6)GalNAcol	Core 2	0.30	√	×	√	*	√
	Mixed acid glycans							

958.2672^{-}	Neu5Acα2-3Galβ1-4(SO_3^-)GlcNAcβ1-3GalNAcol	Core 3	0.34	*	×	×	×	×
974.2620^{-}	Neu5Gcα2-3Galβ1-4(SO_3^-)GlcNAcβ1-3GalNAcol	Core 3	4.53	×	×	√	×	×
982.3572-	Neu5Acα2-3Galβ1-3(NeuGcα2-6)GalNAcol	Core 1	0.17	×	×	×	×	×

'*' means the presence of isomers, '√' means the structures were detected, '×' means the structures were not detected.

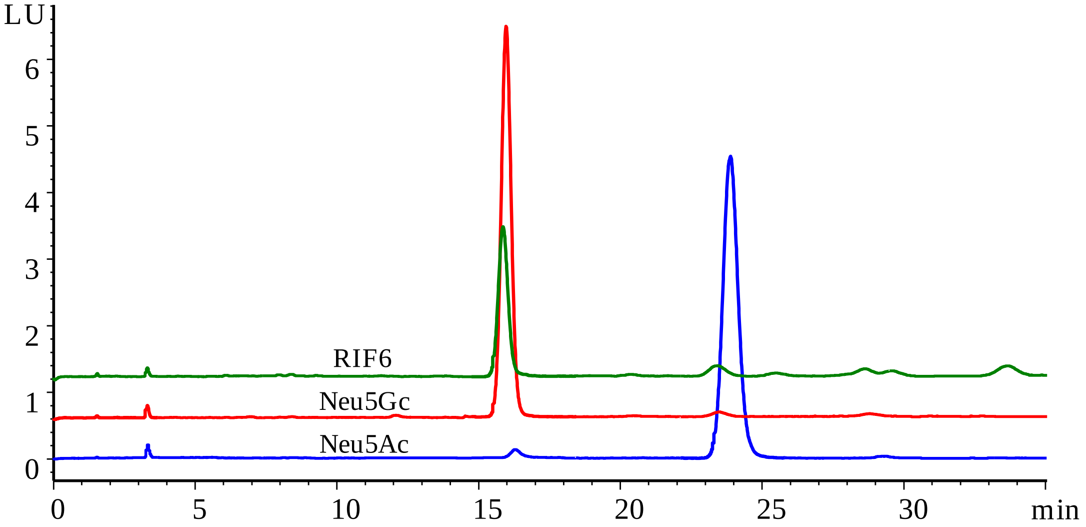

Figure S1. HPLC Chromatogram for sialic acid standards solution (Neu5Ac, Neu5Gc) and sample (RIF6).

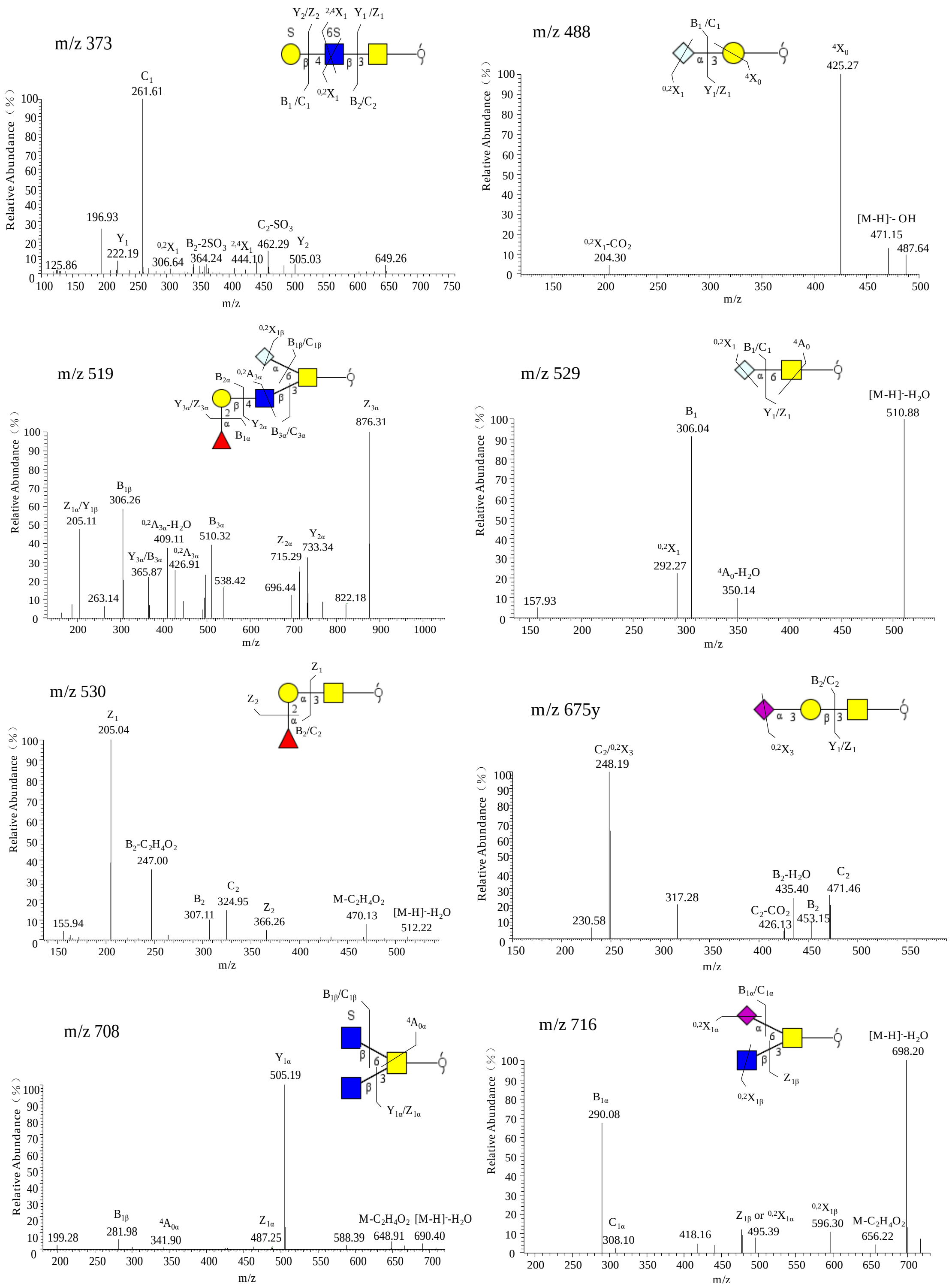

m/z 373
Y2/Z2 2,4X1 Y1/Z1
S 6S
B1/C1 0,2X1 B2/C2
C1 261.61
196.93
Y1 222.19
0,2X1 306.64
B2-2SO3 364.24
2,4X1 444.10
C2-SO3 462.29
Y2 505.03
649.26
125.86
Relative Abundance (%)
m/z
m/z 488
B1/C1
0,2X1 Y1/Z1 4X0
4X0 425.27
0,2X1-CO2 204.30
[M-H]- - OH 471.15
487.64
m/z 519
0,2X1β B1β/C1β
B2α 0,2A3α
Y3α/Z3α Y2α B1α B3α/C3α
Z3α 876.31
B1β 306.26
Z1α/Y1β 205.11
0,2A3α-H2O 409.11
B3α 510.32
Y3α/B3α 365.87
0,2A3α 426.91
Z2α 715.29
Y2α 733.34
263.14
538.42
696.44
822.18
m/z 529
0,2X1 B1/C1 4A0
Y1/Z1
[M-H]--H2O 510.88
B1 306.04
0,2X1 292.27
4A0-H2O 350.14
157.93
m/z 530
Z1
Z2 B2/C2
Z1 205.04
B2-C2H4O2 247.00
B2 307.11
C2 324.95
Z2 366.26
M-C2H4O2 470.13
[M-H]--H2O 512.22
155.94
m/z 675y
B2/C2
0,2X3 Y1/Z1
C2/0,2X3 248.19
317.28
230.58
B2-H2O 435.40
C2 471.46
C2-CO2 426.13
B2 453.15
m/z 708
B1β/C1β
S 4A0α
Y1α/Z1α
Y1α 505.19
B1β 281.98
4A0α 341.90
Z1α 487.25
199.28
588.39
M-C2H4O2 648.91
[M-H]--H2O 690.40
m/z 716
B1α/C1α
0,2X1α Z1β 0,2X1β
[M-H]--H2O 698.20
B1α 290.08
C1α 308.10
418.16
Z1β or 0,2X1α 495.39
0,2X1β 596.30
M-C2H4O2 656.22

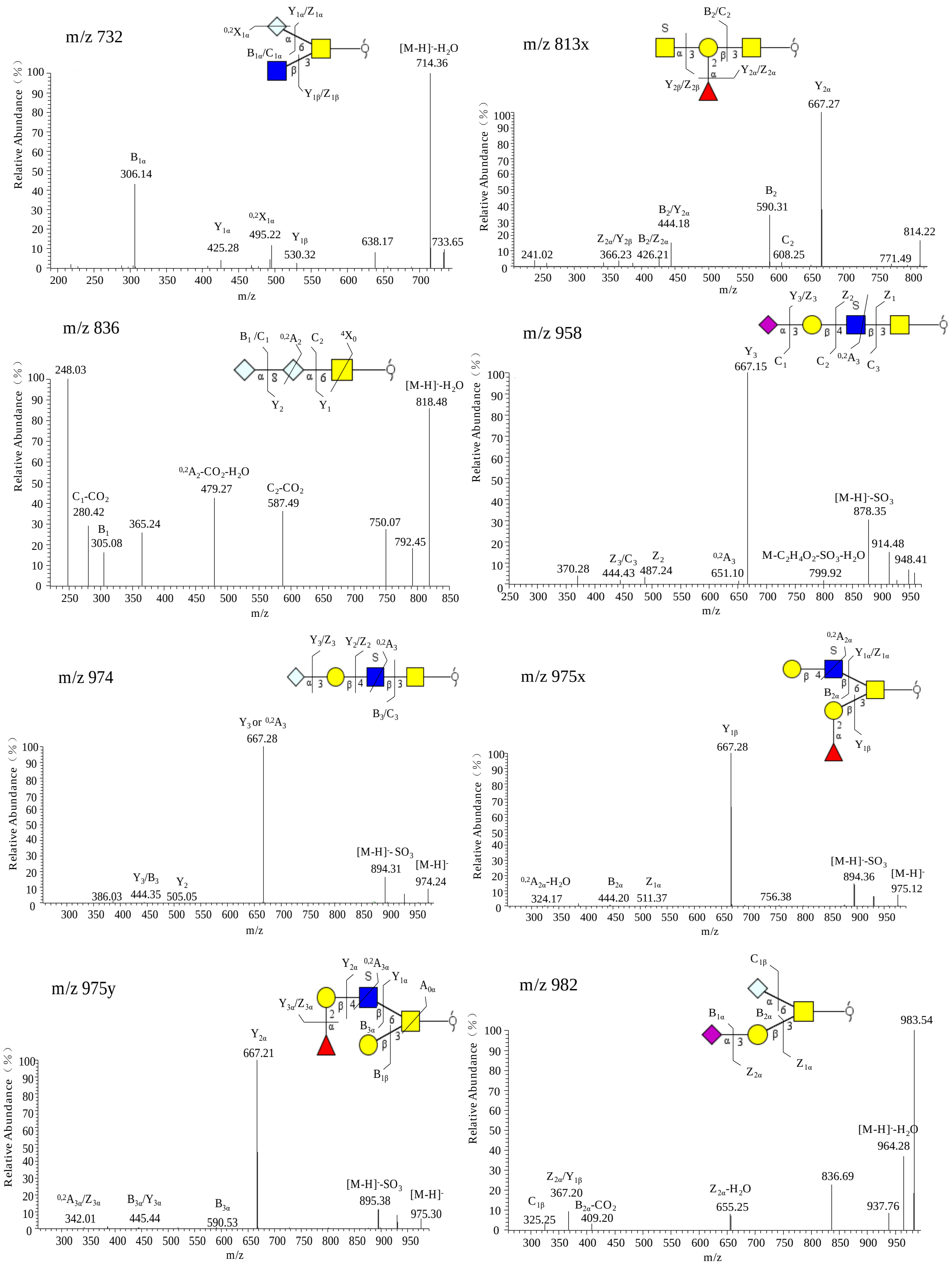
m/z 732
$^{0,2}X_{1\alpha}$
$Y_{1\alpha}/Z_{1\alpha}$
$B_{1\alpha}/C_{1\alpha}$
$Y_{1\beta}/Z_{1\beta}$
$[M-H]^{-}-H_2O$ 714.36
$B_{1\alpha}$ 306.14
$Y_{1\alpha}$ 425.28
$^{0,2}X_{1\alpha}$ 495.22
$Y_{1\beta}$ 530.32
638.17
733.65
Relative Abundance（%）
m/z
m/z 813x
B_2/C_2
S
$Y_{2\beta}/Z_{2\beta}$
$Y_{2\alpha}/Z_{2\alpha}$
$Y_{2\alpha}$ 667.27
B_2 590.31
$B_2/Y_{2\alpha}$ 444.18
$Z_{2\alpha}/Y_{2\beta}$ 366.23
$B_2/Z_{2\alpha}$ 426.21
241.02
C_2 608.25
771.49
814.22
m/z 836
B_1/C_1
$^{0,2}A_2$
C_2
$^4X_0$
Y_2
Y_1
248.03
$[M-H]^{-}-H_2O$ 818.48
C_1-CO_2 280.42
B_1 305.08
365.24
$^{0,2}A_2-CO_2-H_2O$ 479.27
C_2-CO_2 587.49
750.07
792.45
m/z 958
Y_3/Z_3
Z_2
Z_1
S
C_1
C_2
$^{0,2}A_3$
C_3
Y_3 667.15
$[M-H]^{-}-SO_3$ 878.35
914.48
948.41
370.28
Z_3/C_3 444.43
Z_2 487.24
$^{0,2}A_3$ 651.10
$M-C_2H_4O_2-SO_3-H_2O$ 799.92
m/z 974
Y_3/Z_3
Y_2/Z_2
$^{0,2}A_3$
S
B_3/C_3
Y_3 or $^{0,2}A_3$ 667.28
$[M-H]^{-}-SO_3$ 894.31
$[M-H]^{-}$ 974.24
386.03
Y_3/B_3 444.35
Y_2 505.05
m/z 975x
$^{0,2}A_{2\alpha}$
S
$Y_{1\alpha}/Z_{1\alpha}$
$B_{2\alpha}$
$Y_{1\beta}$
$Y_{1\beta}$ 667.28
$[M-H]^{-}-SO_3$ 894.36
$[M-H]^{-}$ 975.12
$^{0,2}A_{2\alpha}-H_2O$ 324.17
$B_{2\alpha}$ 444.20
$Z_{1\alpha}$ 511.37
756.38
m/z 975y
$Y_{2\alpha}$
$^{0,2}A_{3\alpha}$
$Y_{1\alpha}$
$A_{0\alpha}$
$Y_{3\alpha}/Z_{3\alpha}$
S
$B_{3\alpha}$
$B_{1\beta}$
$Y_{2\alpha}$ 667.21
$^{0,2}A_{3\alpha}/Z_{3\alpha}$ 342.01
$B_{3\alpha}/Y_{3\alpha}$ 445.44
$B_{3\alpha}$ 590.53
$[M-H]^{-}-SO_3$ 895.38
$[M-H]^{-}$ 975.30
m/z 982
$C_{1\beta}$
$B_{1\alpha}$
$B_{2\alpha}$
$Z_{2\alpha}$
$Z_{1\alpha}$
983.54
$[M-H]^{-}-H_2O$ 964.28
$Z_{2\alpha}/Y_{1\beta}$ 367.20
$C_{1\beta}$ 325.25
$B_{2\alpha}-CO_2$ 409.20
$Z_{2\alpha}-H_2O$ 655.25
836.69
937.76

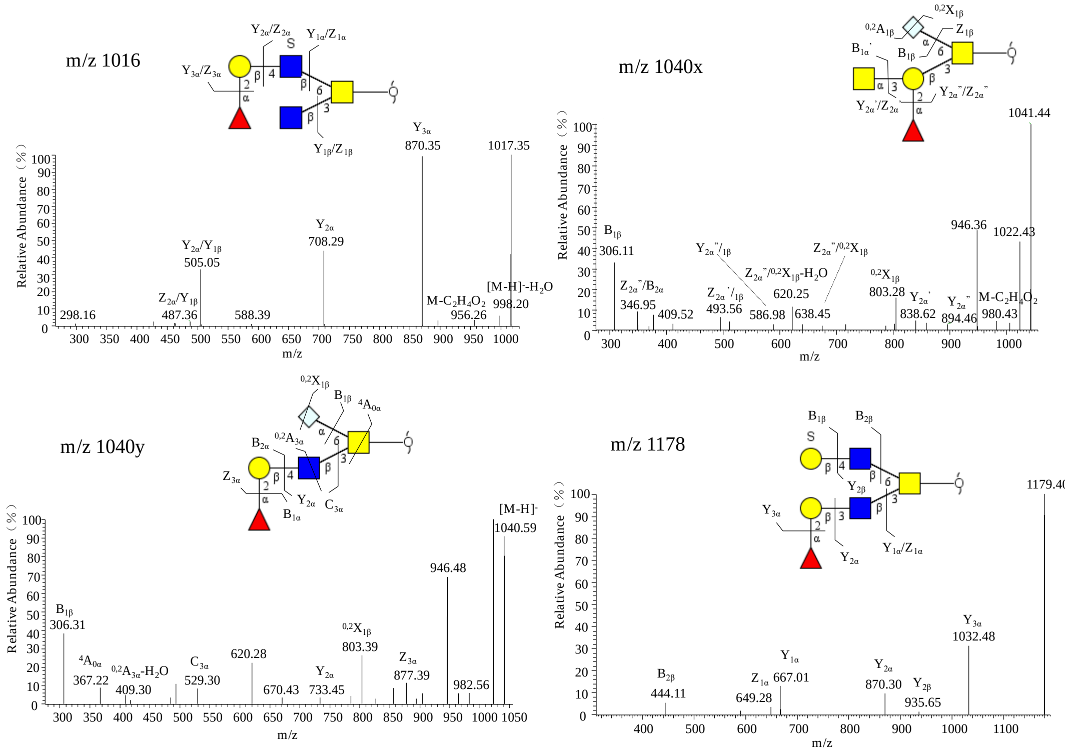

Figure S2. ESI-CID-MS/MS spectra of the remaining ***O***-glycans from RIF6.

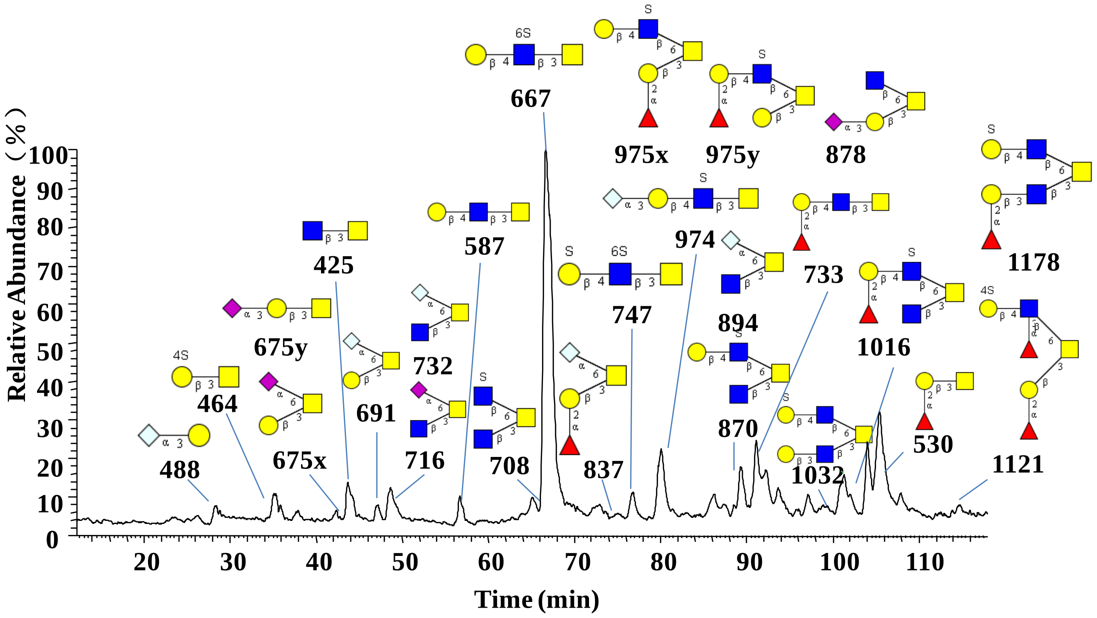

Figure S3. Total ion chromatogram of assigned ***O***-glycan structures of RIF6.
